# Genetic and clinical characteristics of PROM1-related retinal degeneration in Korean

**DOI:** 10.1038/s41598-023-49131-z

**Published:** 2023-12-11

**Authors:** Sungsoon Hwang, Se Woong Kang, Ja-Hyun Jang, Sang Jin Kim

**Affiliations:** 1 Department of Ophthalmology, Samsung Medical Center, Sungkyunkwan University School of Medicine, #81 Irwon-ro, Gangnam-gu, Seoul, 06351 Republic of Korea; 2 Department of Clinical Research Design and Evaluation, Samsung Advanced Institute for Health Sciences and Technology (SAIHST), Sungkyunkwan University, Seoul, Republic of Korea; 3 Department of Laboratory Medicine and Genetics, Samsung Medical Center, Sungkyunkwan University School of Medicine, Seoul, Republic of Korea

**Keywords:** Hereditary eye disease, Retinal diseases

## Abstract

This scientific report aims to comprehensively describe the genetic and clinical characteristics of PROM1-related retinal degeneration in Korean patients. Medical records of patients diagnosed with retinal dystrophy who underwent comprehensive ophthalmologic examination and genetic testing at Samsung Medical Center between January 2016 and April 2023 were retrospectively reviewed. Genetic testing included targeted gene panel sequencing and Sanger sequencing, with diagnosis based on the presence of a “Likely Pathogenic” or “Pathogenic Variant” in the PROM1 gene, as determined by the ACMG criteria. The study identified seven patients from five unrelated families with PROM1-related retinal degeneration, all carrying the autosomal dominant variant PROM1 p.R373C; no other PROM1 gene variants were detected. All patients exhibited degenerative retinal area within the macula, with peripheral retinal degeneration observed in five patients. Substantial interfamilial and intrafamilial variability was observed in the extent of macular and peripheral degeneration. Ultra-widefield autofluorescence imaging and fluorescein angiography aided in the detection of mild peripheral degeneration in one case. In conclusion, the autosomal dominant variant PROM1 p.R373C constitutes a significant proportion of PROM1-related retinal degeneration cases in the Korean population. The observed clinical heterogeneity may suggests the potential influence of additional genetic, epigenetic, and environmental factors on disease phenotypes.

## Introduction

The PROM1 gene encodes the protein prominin-1, a pentaspan transmembrane glycoprotein that is predominantly expressed in a diverse range of stem and progenitor cells, as well as in photoreceptors and retinal pigment epithelia^1,2^. The protein is commonly known by its alternative names, CD133 and AC133, and is widely recognized as a human stem cell-specific surface marker^2^. In the milieu of the retina, this protein exhibits a significant presence within the photoreceptor outer segments and plays a key role in disc morphogenesis^3,4^. More recently, PROM1 has been identified as an integral factor involved in modulating photoreceptor autophagy in the retinal pigment epithelium^5^, underscoring its multi-faceted role in ocular physiology.

Disease-causing variants of PROM1 have been linked to a wide range of retinal phenotypes, including macular dystrophy (MD), cone-rod dystrophy (CRD), and rod-cone dystrophy. Biallelic autosomal recessive variants in PROM1 have been documented to cause retinitis pigmentosa (RP), CRD, or panretinal dystrophy of cone-rod phenotype with early macular involvement^6–11^, while autosomal dominant variant mainly result in Stargardt-like macular dystrophy (STGD4) or CRD with a later onset and a slower decline in visual function^10–17^. The genetic and clinical heterogeneity observed in PROM1-related retinal degeneration highlights the intricate role of the PROM1 gene in the pathomechanism of inherited retinal diseases (IRDs).

Although studies have investigated PROM1-related retinal degeneration in various populations, few have focused on the genetic variants and clinical characteristics of PROM1-associated retinal degeneration within the Asian population^18,19^. Asian populations have a distinct genetic architecture influenced by unique ancestral lineages; therefore, it is crucial to understand the distribution of the variants and their effect on the clinical manifestation of IRDs within this population.

This study aimed to bridge this knowledge gap by investigating the spectrum of genetic variants in PROM1-associated retinal degeneration and to provide an understanding of the overall clinical picture of this condition in the Korean population.

## Methods

### Setting and ethics statement

This was a retrospective study of consecutive patients with PROM1-related retinal degeneration who underwent a comprehensive ophthalmologic examination at the Samsung Medical Center. The study adhered to the tenets of the Declaration of Helsinki and was approved by the Institutional Review Board of the Samsung Medical Center, Seoul, Republic of Korea (IRB number 2023-05-060). The board waived the requirement for informed consent due to the retrospective nature of the study.

### Subjects

The medical records of all consecutive patients with genetically confirmed IRD at the Inherited Retinal Disease Clinic, Department of Ophthalmology, Samsung Medical Center, between January 2016 and April 2023 were retrospectively reviewed. A diagnosis of genetically verified PROM1-related retinal degeneration was established for patients with heterozygous dominant or biallelic recessive variants in PROM1, classified as pathogenic or likely pathogenic, according to the guidelines of the American College of Medical Genetics and Genomics^20^.

### Clinical assessment and imaging

Comprehensive clinical assessments, including medical and family histories, visual acuity measurements, and dilated fundus examinations, were conducted at the Inherited Retinal Disease Clinic. Optical coherence tomography (OCT) images of the macula were obtained using spectral-domain OCT (Spectralis HRA + OCT; Heidelberg Engineering, Heidelberg, Germany). Fundus color photography (TRC 50DX; Topcon, Tokyo, Japan) with autofluorescence (Spectralis HRA + OCT; Heidelberg Engineering, Heidelberg, Germany) or ultra-widefield fundus photography with fundus autofluorescence (FAF) (Optos 200Tx; Optos Plc, Dunfermline, Scotland, UK) was also conducted routinely. Full-field electroretinography (RETIScan system; Roland Consult, Wiesbaden, Germany) was performed according to the protocol proposed by the international standards of the International Society for Clinical Electrophysiology of Vision^21^.

### Genetic testing

Genomic DNA was extracted from the peripheral blood of patients with IRD. Targeted gene panel sequencing using next-generation sequencing (NGS) technique was carried out using the Illumina HiSeq 2500 or NextSeq 550Dx platform (Illumina, San Diego, CA, USA). All variants identified by NGS were confirmed by Sanger sequencing. In patients having family members who had already been genetically diagnosed with IRD, direct Sanger sequencing for the identified variant was performed.

## Results

Among patients with genetically confirmed IRD who visited the Inherited Retinal Disease Clinic, seven patients from five families with PROM1-related retinal degeneration were identified. All patients diagnosed with PROM1-related retinal degeneration harbored the heterozygous missense variant p.R373C (NM_006017.2:c.1117C > T), which presented an autosomal dominant inheritance pattern. Two patients were male and five were female, and the median age of the subjects was 37 years (range: 30–70 years). All patients exhibited degeneration within the macula with various degrees of severity, and five patients presented with peripheral retinal degeneration. No abnormalities were observed in the anterior segment in any patient. The participant demographic characteristics, histories, and clinical findings are summarized in the Table 1. Pedigrees of the patients are presented in supplemental Fig. 1. Some data from one patient (P1) has been published elsewhere^13^.

**Table 1:** Demographic and clinical characteristics of Korean patients with PROM1-related retinal degeneration.

Patient no.	Family no.	Variant	Age	Presenting symptoms	Age at onset	Visual acuity	Ocular phenotype	Macular findings	Peripheral findings	Electrophysiologic findings
1	1	p.R373C	37	DV	4th decade	0.6 OU	Mainly MD/CRD	BEM OU	No available data	Rod: moderately decreased Cone: moderately decreased
2	2	p.R373C	34	DV	3rd decade	0.15 OD 0.5 OS	Mainly MD/CRD	GA OD BEM OS	No available data	Rod: moderately decreased Cone: moderately decreased
3	3	p.R373C	32	–	–	1.0 OU	Mainly MD/CRD	BEM OU	Mild degeneration without BSP	Rod: mildly decreased Cone: moderately decreased
4	3	p.R373C	30	–	–	1.0 OU	Mainly RP	BEM OU	Degeneration with BSP	Rod: extinguished Cone: severely decreased
5	4	p.R373C	70	NB	3rd decade	1.0 OU	Mainly RP	BEM OU	Degeneration with BSP	Not available
6	4	p.R373C	40	DV, NB	4th decade	0.6 OD 0.5 OS	Mainly MD/CRD	BEM OU	Degeneration with BSP	Rod: extinguished Cone: severely decreased
7	5	p.R373C	40	DV	4th decade	0.4 OD 0.1 OS	Mainly MD/CRD	BEM OU	Mild degeneration without BSP	Rod: moderately decreased Cone: moderately decreased

*BEM* bull’s eye maculopathy, *BSP* bone spicule pigmentation, *CRD* cone-rod dystrophy, *DV* decreased visual acuity, *GA* geographic atrophy, *MD* macular dystrophy, *NB* night blindness, *OD* oculus dexter, *OS* oculus sinister, *OU* oculus uterque, *RP* retinitis pigmentosa.

### Patient P1

**Figure 1:**
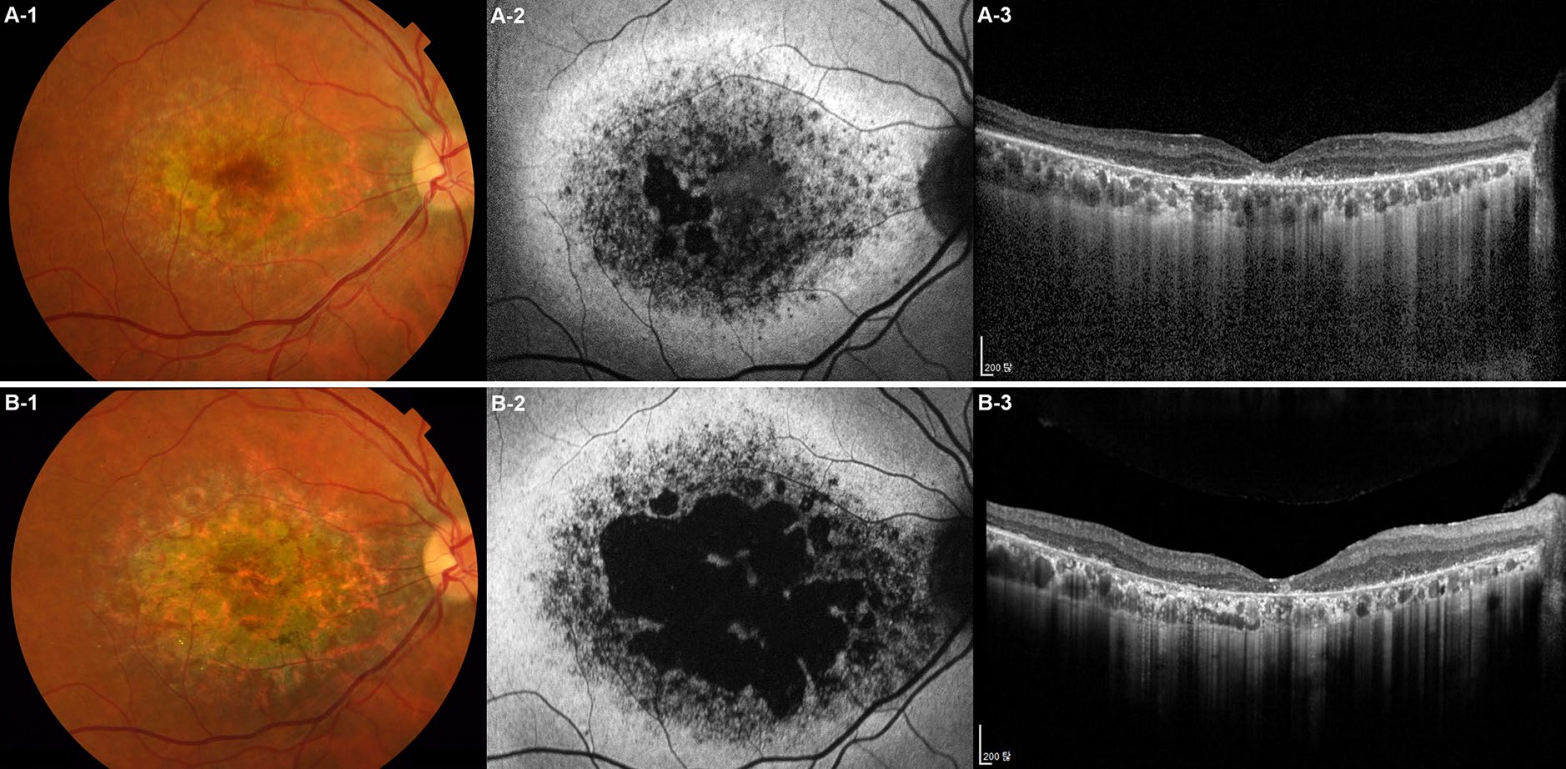
Longitudinal progression of PROM1 p.R373C-associated retinal degeneration. A 37-year-old male patient (P1) was followed up for 7 years. (**A-1**) Initially, the color fundus photograph revealed a moderate degree of macular degeneration, (**A-2**) and the fundus autofluorescence (FAF) image revealed bull’s eye maculopathy with a central area of heterogeneous autofluorescence (AF) surrounded by a hyper-AF ring. (**A-3**) The optical coherence tomography (OCT) revealed disruptions in the outer retinal layer and retinal pigment epithelium atrophy at the temporal parafoveal area, corresponding to the small area of geographic atrophy observed in the FAF image. By the age of 44, the visual acuity of the patient had deteriorated from 0.6 to 0.1 in both eyes. (**B-1**) A follow-up color fundus photo and (**B-2**) FAF revealed a significant enlargement of the geographic atrophy area, now encompassing almost the entire macular region, indicating disease progression. (**B-3**) Subsequent OCT imaging demonstrated a profound degeneration of the outer retinal layer with extensive loss of the retinal pigment epithelium, consistent with the advanced disease stage.

A 37-year-old male patient (P1) harboring the PROM1 p.R373C variant presented with decreased visual acuity that had commenced a few years before. The best-corrected visual acuity was 0.6 in both eyes. Color fundus photography and FAF revealed bull’s eye maculopathy with an area of heterogeneous autofluorescence (AF) surrounded by a hyper-AF ring. OCT revealed disruption of the outer retinal layer and retinal pigment epithelium atrophy (Fig. 1A). Seven years later, visual acuity had harshly deteriorated to 0.1 in both eyes. The bull’s eye maculopathy progressed to central geographic atrophy encompassing the entire macula (Fig. 1B).

### Patients P3 and P4

**Figure 2:**
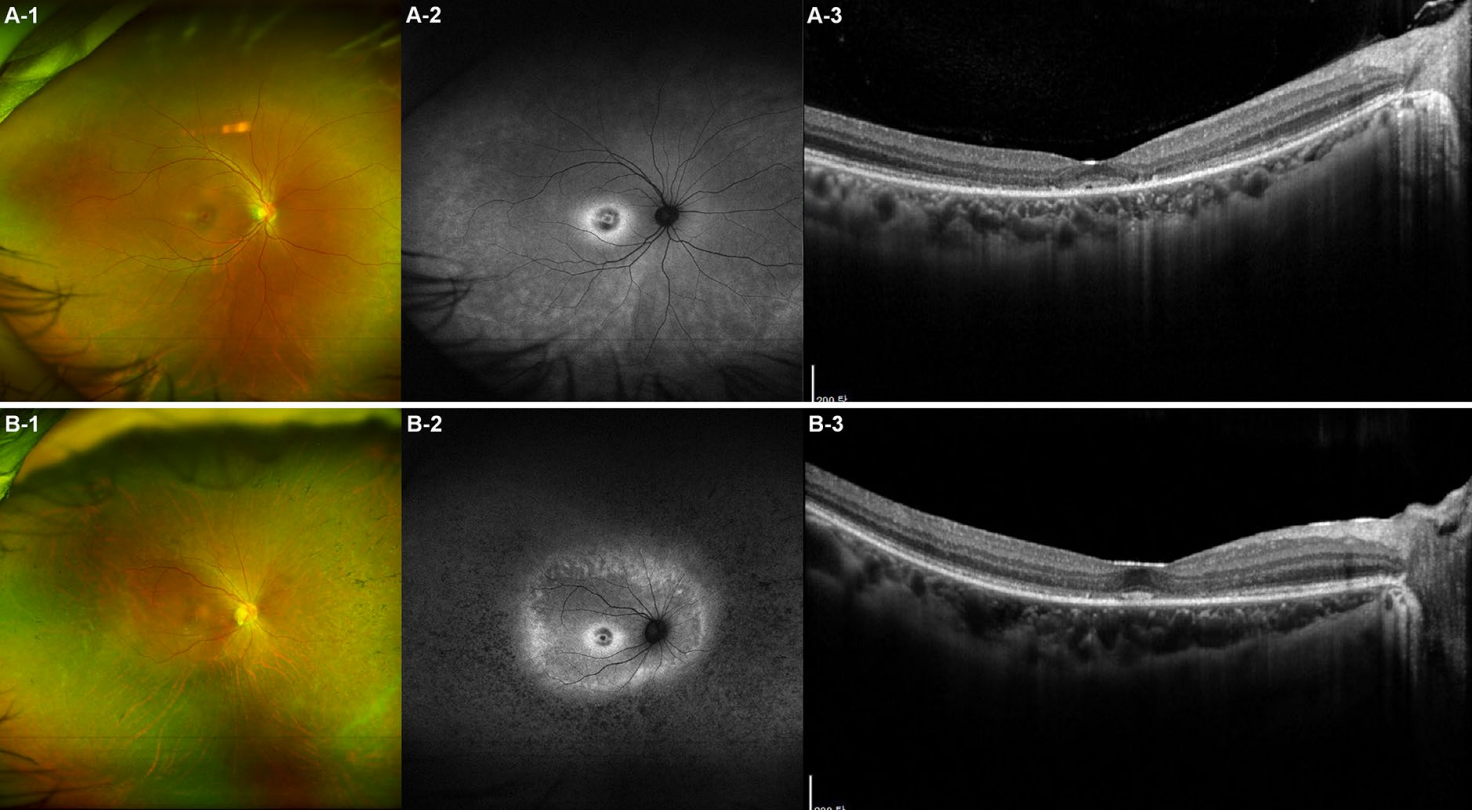
Intrafamilial variability in clinical findings of PROM1 p.R373C-associated retinal degeneration observed in two siblings. The two patients, denoted as patient P3 and P4, are siblings, with P3 being the elder sister aged 32 years, and P4 being the younger sister aged 30 years. (**A-1**) The ultra-widefield fundus photo of P3 reveals mild degeneration at the macula and mild tapetoretinal degeneration in the periphery. (**A-2**) The ultra-widefield fundus autofluorescence (FAF) image illustrates a hyper-autofluorescence (AF) ring in the macula and a mild mosaic pattern of hyper-AF distributed in the peripheral retina. (**A-3**) Optical coherence tomography (OCT) demonstrates outer retinal layer degeneration, primarily affecting the parafoveal region. (**B-1**) The ultra-widefield fundus photo of P4 illustrates diffuse peripheral retinal degeneration with bony-spicule pigments. (**B-2**) The ultra-widefield FAF image shows a hyper-AF ring at the macula, similar to that observed in P3. Additionally, there is a loss of AF in the peripheral retina surrounding a second, larger hyper-AF ring, observed slightly outside the vascular arcade. (**B-3**) The OCT demonstrates parafoveal outer retinal degeneration, similar to that observed in P3. However, the foveal photoreceptor and outer nuclear layers in P4 appear relatively better preserved than those in P3.

**Figure 3:**
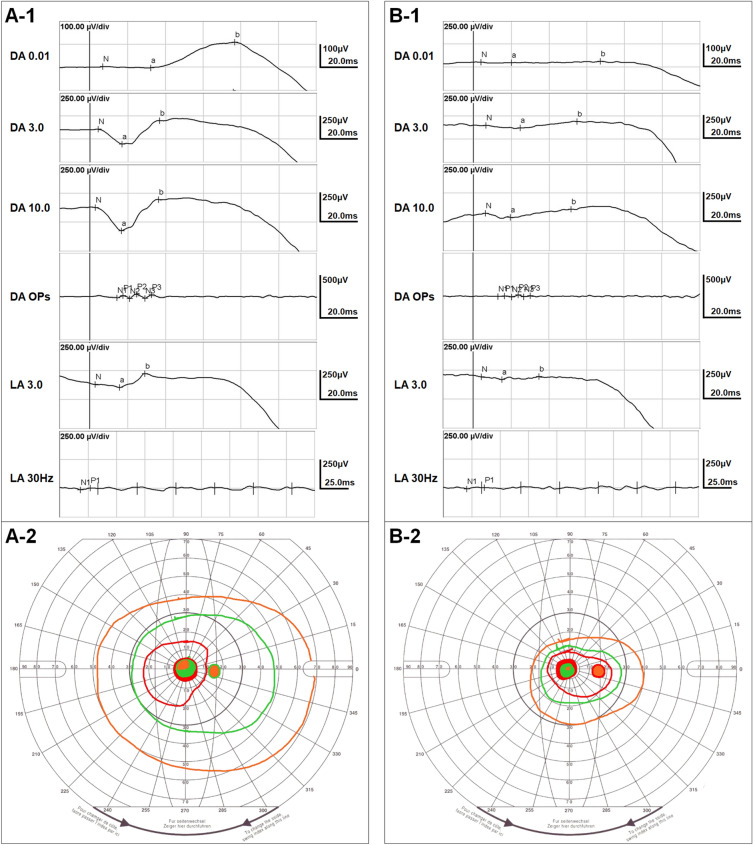
Intrafamilial variability in retinal function of PROM1 p.R373C-associated retinal degeneration observed in two siblings. The two siblings, designated as patient P3 and P4 underwent electroretinography and Goldman visual field tests. (**A-1**) Electroretinography of P3 reveals mildly decreased rod function with moderately decreased cone function in the retina. (**B-1**) In contrast, electroretinography of P4 illustrates extinguished rod function and severely decreased cone function in the retina. The Goldman visual field test displays the results using orange, green, and red lines for the III4e, I3e, and I2e targets, respectively. (**A-2**) In P3, the Goldman visual field test demonstrates a normal visual field. (**B-2**) In contrast, the Goldman visual field test of P4 shows a significantly more constricted visual field compared to that of P3. These findings are consistent with the imaging findings for P3 and P4, depicted in Fig. 2.

P3 and P4 were siblings, with P3 being the elder sister, aged 32, and P4 being the younger sister, aged 30. They were diagnosed with PROM1 p.R373C-associated retinal degeneration after undergoing a comprehensive examination for incidental retinal abnormalities. These two sisters exhibited relatively similar phenotypes in the macula. Both patients had a best-corrected visual acuity of 1.0 in both eyes, and similar pattern of degeneration at macula with a hyper-AF ring and parafoveal outer retinal degeneration were observed by ultra-widefield FAF and OCT. However, they exhibited distinct phenotypic differences in the peripheral retina. P3 exhibited only a mild tapetoretinal degeneration on ultra-widefield fundus photography and mild mosaic hyper-AF on ultra-widefield FAF, while P4 presented severe peripheral retinal degeneration and bony spicules (Fig. 2). Functional testing also revealed substantial differences between the patients. Specifically, P3 showed mildly decreased rod responses on electroretinography, whereas P4 showed a nearly diminished rod response. Additionally, only P4 exhibited significant visual field constriction in Goldman kinetic perimetry (Fig. 3).

### Patients P5 and P6

**Figure 4:**
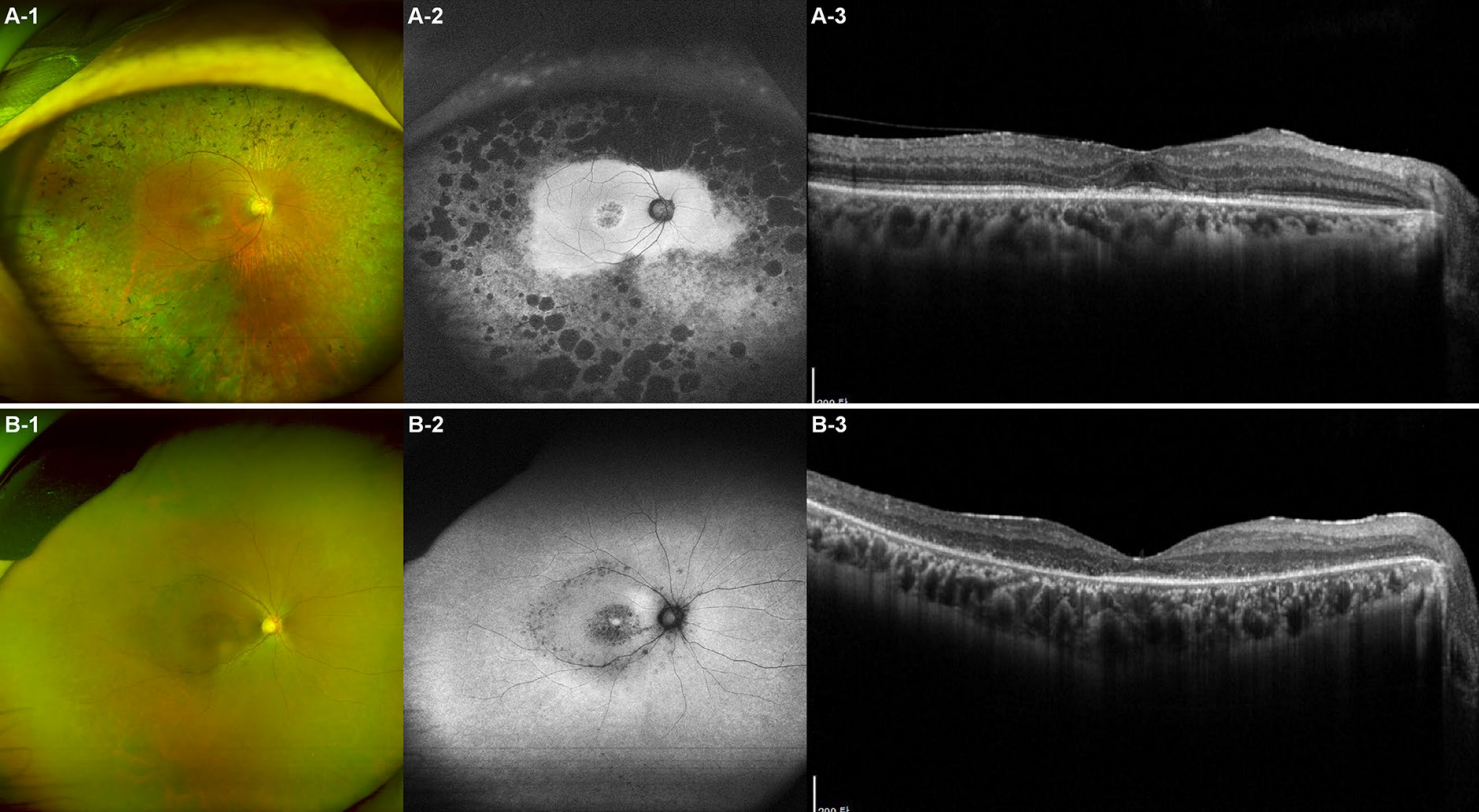
Intrafamilial variability in clinical findings of PROM1 p.R373C-associated retinal degeneration observed in a father-daughter pair. The two patients, identified as P5 and P6, are father and daughter, respectively, with P5 being 70 years old, and P6 being 40 years old. (**A-1**) The ultra-widefield fundus photo of the father, P5, reveals mild degeneration in the macula and extensive peripheral retinal degeneration with bony-spicule pigmentation. (**A-2**) The ultra-widefield fundus autofluorescence (FAF) image illustrates a central area of heterogeneous autofluorescence (AF) surrounded by a hyper-AF ring at the macula, along with extensive loss of AF and nummular retinal pigment epithelium atrophy outside the vascular arcade. (**A-3**) The optical coherence tomography (OCT) photo demonstrates mild parafoveal degeneration with preserved external limiting membrane and retinal pigmented epithelium. (**B-1**) The ultra-widefield fundus photo of the daughter, P6, illustrates diffuse macular and peripheral retinal degeneration with mild bony-spicule pigments along the major vascular arcade. (**B-2**) Ultra-widefield FAF shows a central area of heterogeneous AF, which is more severe than that observed in her father, P5. AF is relatively preserved except for the macula and the area along the vascular arcade. (**B-3**) The OCT demonstrates outer retinal degeneration in the entire macular area, with the photoreceptor layer only visible at the fovea. The outer retinal degeneration at the macula is much more severe compared to that in P5.

P5 and P6 were a father-daughter pair, with P5 being the 70-year-old father and P6 being the 40-year-old daughter. P5 visited the clinic because of nyctalopia, whereas P6 sought consultation for decreased visual acuity and nyctalopia; both patients were subsequently diagnosed with PROM1 p.R373C-associated retinal degeneration. Interestingly, they exhibited different phenotypes in the macular area. P5 had a best-corrected visual acuity of 1.0 in both eyes, whereas P6 had a measurement of 0.6 in her right eye and 0.5 in her left eye. FAF imaging revealed a noticeably wider area of degeneration at the macula in P6. Additionally, OCT revealed that P5 displayed very mild parafoveal degeneration, whereas P6 exhibited extensive outer retinal degeneration in the macular area (Fig. 4). Considering their age, P5 demonstrated a significantly milder macular phenotype. Regarding peripheral retinas, both patients displayed wide-spread peripheral retinal degeneration, with the father exhibiting a more pronounced retinal degeneration at the periphery.

## Discussion

The present study elucidated a high proportion of autosomal dominant variants PROM1 p.R373C in Korean PROM1-associated retinal degeneration, in Korean cases of PROM1-associated retinal degeneration, along with a substantial degree of interfamilial and intrafamilial phenotypic variability in PROM1 p.R373C-associated retinal degeneration. This high phenotypic variability underscores the intricate nature of PROM1 p.R373C variant-associated retinal disease and suggests the probable influence of additional genetic, epigenetic, or environmental modifiers on the clinical phenotype.

The PROM1 p.R373C variant has been reported to be predominant in autosomal dominant PROM1-related IRD^11,15^, although some other autosomal dominant PROM1 variants have recently been identified^10,22^. Reports from European countries have indicated that autosomal dominant cases constitute less than one-third of all PROM1-related IRD cases^10,11^. In contrast, the p.R373C variant was the only pathogenic or likely pathogenic PROM1 variant identified in our study, and no other variants of the PROM1 gene were detected, highlighting a significant difference in the genetic spectrum of the PROM1 variants. To date, there have been no reports of autosomal recessive PROM1-related IRD in Korea. Moreover, in a previous genetic study on IRD conducted at a single tertiary hospital, three PROM1 patients were reported, all of whom carried the p.R373C variant^23^. These findings indicate a considerable prevalence of autosomal dominant PROM1 p.R373C variants within Korean patients of PROM1-related IRD. Interestingly, according to previous reports, the p.R373C variant was found in six out of eight Japanese families with PROM1-related IRD^18^ and in four out of seven Chinese families with PROM1-related IRD^19^, which closely aligns with the results of the present study. These findings suggest a higher proportion of autosomal dominant PROM1 p.R373C variants in Asian populations than in Western populations.

In this study, significant diversity in ocular presentation was observed, despite all patients having the same genetic variant. The onset of symptoms uniformly occurred in the 3rd or 4th decade across all subjects, yet their presenting symptoms varied widely. Night blindness, decreased visual acuity, or a combination of both symptoms were encountered in different cases. Notably, even within the same family, substantial differences in the severity of macular degeneration were evident between patients P5 and P6, and similarly, patients P3 and P4 exhibited significant disparities in peripheral degeneration. The severity of the phenotype manifestation, whether more pronounced in the macula or periphery, appears to be a decisive factor in clinical presentation. When peripheral degeneration is dominant, it may present as RP, whereas when the macula is predominantly affected, it may manifest as MD or CRD. This observation aligns with previous reports where the PROM1 p.R373C variant has been associated with various phenotypes such as MD^13,14,17^, CRD^11,15^, or RP^10,15^. The phenotypic heterogeneity associated with PROM1 p.R373C has been consistently reported in both Asian and Western populations^15,17–19^. In conclusion, the key clinical features associated with the PROM1 R373C variant can be summarized as varied type of macular dystrophy combined with variable degree of peripheral degeneration.

**Figure 5:**
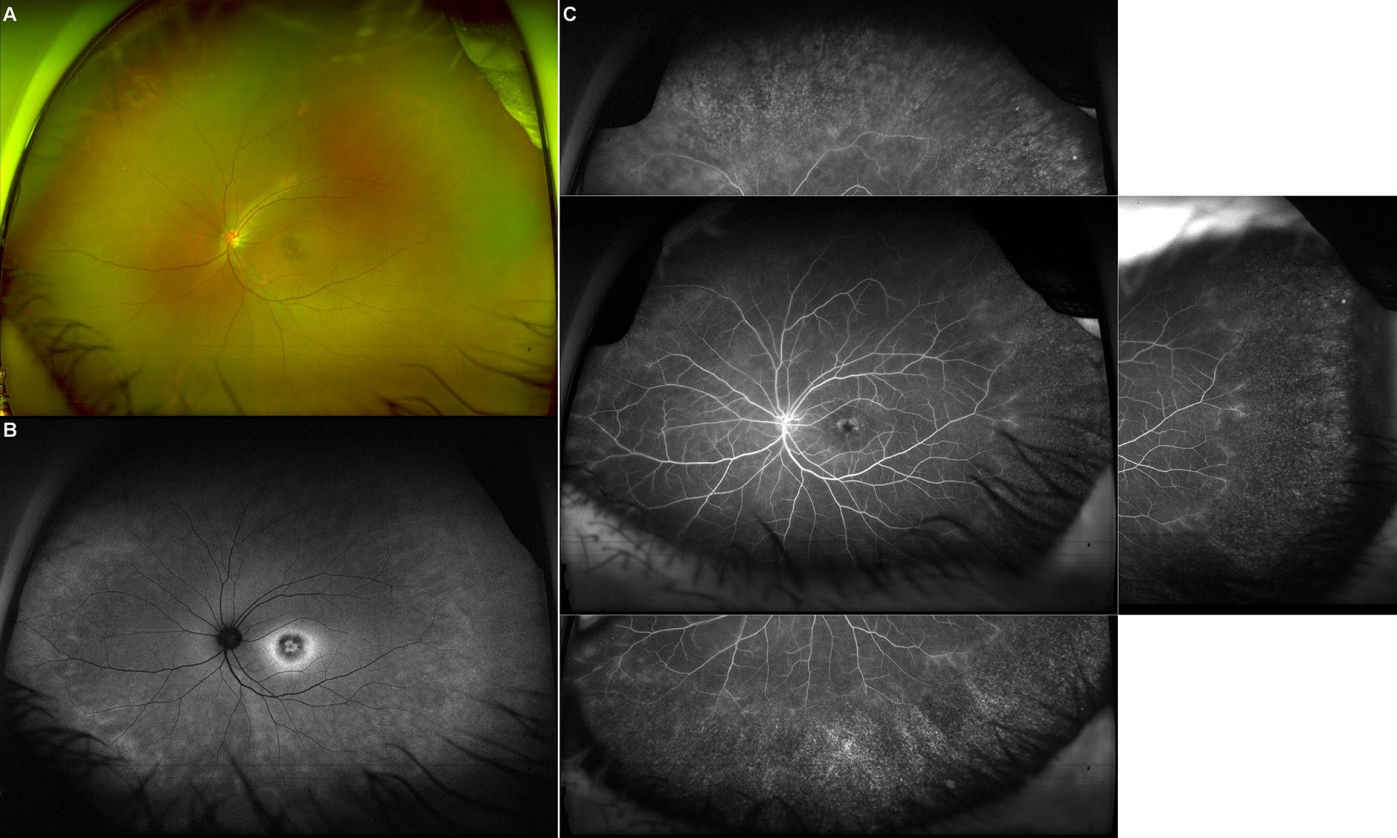
Ultra-widefield fundus imaging of peripheral retinal changes associated with PROM1-related retinal degeneration. (**A**) The ultra-widefield fundus photograph of P3 presents only subtle peripheral retinal changes. (**B**) Ultra-widefield fundus autofluorescence illustrates a mild reticular pattern of hyperautofluorescence distributed in the periphery, which may be indicative of degenerative processes occurring in the peripheral retina. (**C**) Ultra-widefield fundus fluorescein angiography shows obvious peripheral retinal degeneration with vascular attenuation. Mild vascular leakage was also observed at the border of the peripheral retinal degeneration.

In cases of mild macular involvement (as observed in patients P3, P4, and P5), visual acuity remained within the normal range of 1.0. Nevertheless, these patients exhibited parafoveal photoreceptor degeneration patterns that were clearly distinguishable on OCT. When considering peripheral degeneration, ultra-widefield fundus photography alone was able to reveal evident degeneration with extensive bone spicule pigmentation in severe cases. However, in milder cases, abnormalities were not readily apparent through ultra-widefield fundus photography alone, making it necessary to utilize ultra-widefield FAF or ultra-widefield fundus fluorescein angiography (FFA) for accurate detection (Fig. 5). We did not find any evidence of peripheral retinal degeneration in P1 or P2. In the case of P1, ultra-widefield fundus photography was not performed, and in the case of P2, although ultra-widefield fundus photography was performed, no clear abnormalities were observed. If ultra-widefield FAF and FFA had been performed for P1 and P2, peripheral retinal degeneration might have been detected. In a previous study, it was reported that peripheral degeneration was absent in all patients with the PROM1 p.R373C variant^11^. However, this observation could be attributed to the degeneration not being severe enough and the omission of ultra-widefield fundus photography and FAF examinations. In contrast, another study confirmed the presence of peripheral retinal degeneration in all patients with the PROM1 p.R373C variant^19^. Taken together, these findings suggest that even in patients with autosomal dominant PROM1 presenting with MD and related symptoms, ultra-widefield fundus photography, FAF, or, in some instances, FFA, could be advantageous for evaluating and detecting concurrent peripheral degeneration.

PROM1 p.R373C manifested as various phenotypes, even within a family, and the underlying pathomechanism of this heterogeneity remains poorly understood. Phenotypic heterogeneity has also been reported in cases of autosomal recessive PROM1-related retinal degeneration^10^. PROM1 is primarily concentrated in the photoreceptor outer segment disc membranes and is traditionally believed to play a crucial role in the structural organization of the photoreceptor outer segment^4^. The p.R373C missense mutation introduces an additional cysteine residue in the predicted first extracellular loop of prominin-1, thereby disrupting a network of disulfide bridges and consequently impairing homophilic protein interactions^3^. This disruption results in the mislocalization of the variant PROM1 protein, which further affects the localization of the wild-type PROM1 protein and interferes with the function of PCDH21, causing a dominant negative effect^3^. Additionally, PROM1 plays a significant role in the regulation of the visual cycle. When PROM1 is knocked out in the retina, there is a substantial reduction in the expression of visual cycle components, RDH12, and ABCA4^24,25^. Moreover, photoreceptor cell degeneration in the PROM1-knockout retina appears to be light-dependent^24^. Considering these findings, it is speculated that PROM1-related retinal degeneration may be influenced by interactions with other genes, epigenetic factors, or environmental influences, which could explain the observed phenotypic heterogeneity.

Determining the precise pathomechanism of PROM1-related retinal degeneration is vital for the future development and implementation of potential therapeutic options, such as gene therapy. For autosomal recessive PROM1-related IRD, subretinal delivery of adeno-associated viral vectors carrying the PROM1 gene to photoreceptors is a viable approach that may not rely on specific variants. For autosomal dominant PROM1-related IRD, efforts are needed to silence the corresponding variant using antisense oligonucleotides or correct it using CRISPR technology. Fortunately, the majority of autosomal dominant PROM1-related IRD cases involve the p.R373C variant, which provides the advantage of focusing on developing therapies targeting this specific mutation. Nevertheless, the phenotypic diversity of this genetic alteration may present challenges in determining surrogate outcome measures for future gene therapy development and therapeutic trials. Therefore, a comprehensive natural history study to observe detailed phenotypic changes during longitudinal progression is imperative^14^. This will facilitate the establishment of appropriate outcome measures to guide the development of potential therapies for PROM1-related IRD.

Limitations of this study include the limited number of patients and its retrospective nature. Therefore, a multicenter prospective study with a well-structured and standardized ophthalmic examination protocol is warranted.

In conclusion, the autosomal dominant variant PROM1 p.R373C accounts for a significant proportion of all PROM1-related retinal degeneration cases in the Korean population. The extent of degeneration it the macula and periphery varies substantially both between and within families, resulting in the potential manifestations of MD, CRD, or RP phenotypes. This complexity highlights the intricate nature of this genetic condition and suggests that external factors beyond the PROM1 gene may contribute to the clinical presentation, necessitating further research to identify and understand their implications. Furthermore, ultra-widefield imaging, especially FAF and FFA, may be beneficial for the detection of mild peripheral degenerative changes in retinal degeneration caused by the PROM1 p.R373C variant.

### Supplementary Information

Supplementary Figure 1.
