# Supplementary material for: Genetic and clinical characteristics of PROM1-related retinal degeneration in Korean
Source: Sci Rep. 2023 Dec 11;13:21877. doi: 10.1038/s41598-023-49131-z (PMC10711002; doi:10.1038/s41598-023-49131-z)

Genetic and Clinical Characteristics of PROM1-related Retinal Degeneration in Korean

Sungsoon Hwang, MD[1,2]; Se Woong Kang, MD, PhD[1]; Ja-Hyun Jang, MD, PhD[3]; Sang Jin Kim, MD, PhD[1]

[1]Department of Ophthalmology, Samsung Medical Center, Sungkyunkwan University School of Medicine, Seoul, Republic of Korea

[2]Department of Clinical Research Design and Evaluation, Samsung Advanced Institute for Health Sciences and Technology (SAIHST), Sungkyunkwan University, Seoul, Republic of Korea

[3]Department of Laboratory Medicine and Genetics, Samsung Medical Center, Sungkyunkwan University School of Medicine, Seoul, Republic of Korea

Correspondence

Sang Jin Kim, MD, PhD

Department of Ophthalmology, Samsung Medical Center, Sungkyunkwan University School of Medicine, #81 Irwon-ro, Gangnam-gu, Seoul 06351, Republic of Korea

Tel: 82-2-3410-3548, Fax: 82-2-3410-0074

E-mail: sangjin.kim.md@gmail.com

Supplemental Figure 1. Pedigrees of seven patients from five families of PROM1-related inherited retinal disease.

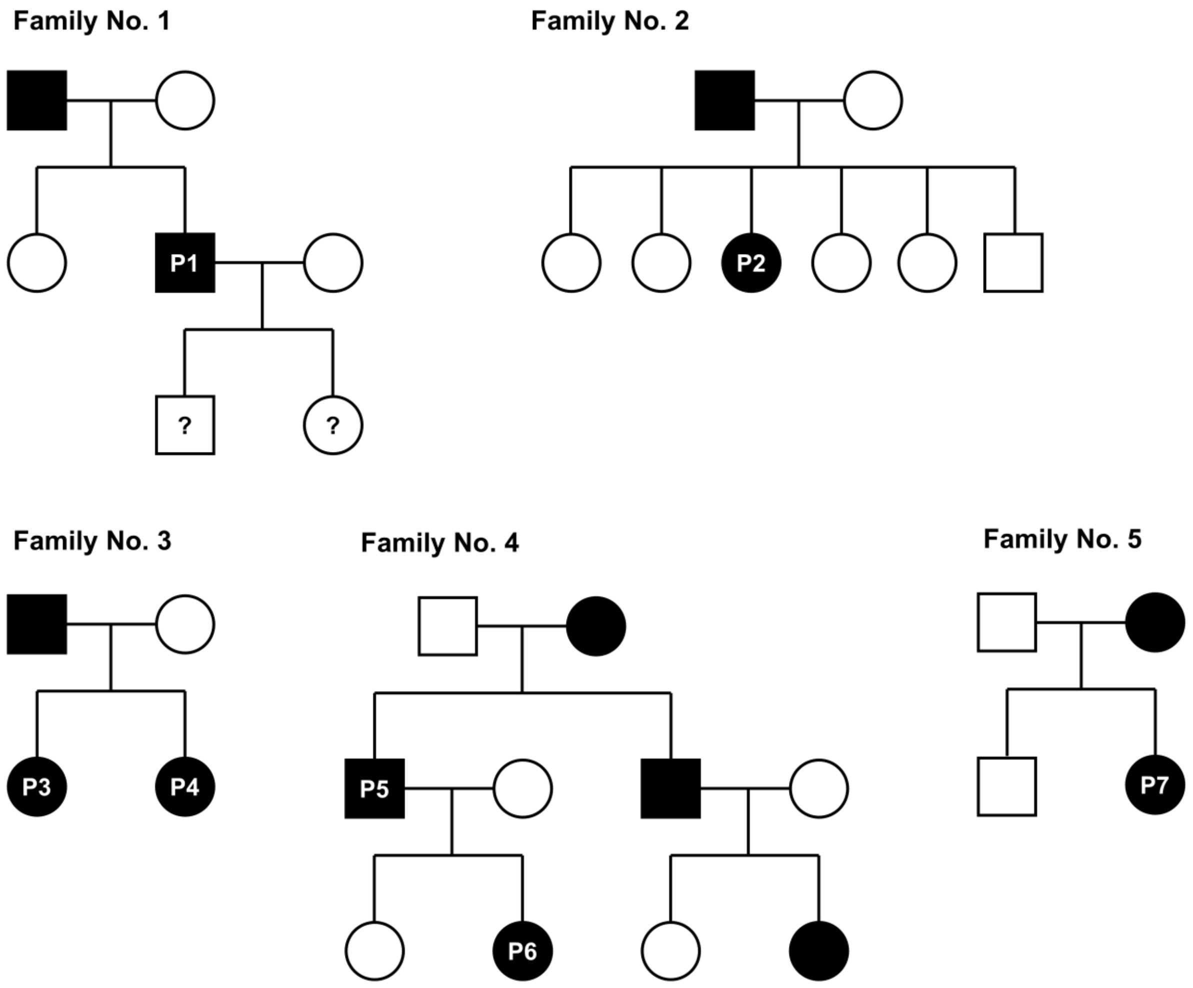

Supplement: Supplementary file 1 — Supplementary Figure 1. [file 41598_2023_49131_MOESM1_ESM.pdf]
